# Computed tomography radiomic features hold prognostic utility for canine lung tumors: An analytical study

**DOI:** 10.1371/journal.pone.0256139

**Published:** 2021-08-17

**Authors:** Hannah Able, Amber Wolf-Ringwall, Aaron Rendahl, Christopher P. Ober, Davis M. Seelig, Chris T. Wilke, Jessica Lawrence

**Affiliations:** 1 Department of Veterinary Clinical Sciences, College of Veterinary Medicine, University of Minnesota, Saint Paul, Minnesota, United States of America; 2 Masonic Cancer Center, University of Minnesota, Minneapolis, Minnesota, United States of America; 3 Department of Veterinary and Biomedical Sciences, College of Veterinary Medicine, University of Minnesota, Minneapolis, Minnesota, United States of America; 4 Department of Radiation Oncology, Medical School, University of Minnesota, Minneapolis, Minnesota, United States of America; Colorado State University, UNITED STATES

## Abstract

Quantitative analysis of computed tomography (CT) radiomic features is an indirect measure of tumor heterogeneity, which has been associated with prognosis in human lung carcinoma. Canine lung tumors share similar features to human lung tumors and serve as a model in which to investigate the utility of radiomic features in differentiating tumor type and prognostication. The purpose of this study was to correlate first-order radiomic features from canine pulmonary tumors to histopathologic characteristics and outcome. Disease-free survival, overall survival time and tumor-specific survival were calculated as days from the date of CT scan. Sixty-seven tumors from 65 dogs were evaluated. Fifty-six tumors were classified as primary pulmonary adenocarcinomas and 11 were non-adenocarcinomas. All dogs were treated with surgical resection; 14 dogs received adjuvant chemotherapy. Second opinion histopathology in 63 tumors confirmed the histologic diagnosis in all dogs and further characterized 53 adenocarcinomas. The median overall survival time was longer (p = 0.004) for adenocarcinomas (339d) compared to non-adenocarcinomas (55d). There was wide variation in first-order radiomic statistics across tumors. Mean Hounsfield units (HU) ratio (p = 0.042) and median mean HU ratio (p = 0.042) were higher in adenocarcinomas than in non-adenocarcinomas. For dogs with adenocarcinoma, completeness of excision was associated with overall survival (p<0.001) while higher mitotic index (p = 0.007) and histologic score (p = 0.037) were associated with shorter disease-free survival. CT-derived tumor variables prognostic for outcome included volume, maximum axial diameter, and four radiomic features: integral total, integral total mean ratio, total HU, and max mean HU ratio. Tumor volume was also significantly associated with tumor invasion (p = 0.044). Further study of radiomic features in canine lung tumors is warranted as a method to non-invasively interrogate CT images for potential predictive and prognostic utility.

## Introduction

Lung cancer is the most commonly diagnosed cancer and is a leading cause of human cancer death worldwide [1–3]. There are two major categories of human lung cancer, namely small cell lung cancer (SCLC) and non-small cell lung cancer (NSCLC). The latter is most common and can be further subdivided into adenocarcinoma and squamous cell carcinoma. It has become increasingly important to accurately identify and classify lung cancer prior to devising a treatment strategy [4–8]. The standard method of classification is via histopathological evaluation of biopsy samples [3]. However, obtaining a lung biopsy is invasive, associated with a risk of minor and major complications, expensive, and requires time for histopathologic review [3, 9]. The identification of a semi-automated, quantitative imaging method by which lung tumors could be non-invasively and accurately classified may provide important predictive information to positively impact clinical practice [10, 11].

Canine cancer is a recognized model for many human tumor types, given the diagnostic and treatment strategies are similar across species [12, 13]. Like humans, older dogs are at risk for development of spontaneous lung tumors, with primary lung tumors accounting for up to 9% of diagnosed tumors in dogs [14, 15]. Also paralleling humans, canine lung tumors may be identified incidentally on thoracic imaging during screening or during investigation of respiratory or other non-specific symptoms [2, 3, 16–19]. Diagnosis is made through histopathologic evaluation, while prognostic and therapeutic recommendations often rely on both diagnostic and histopathologic data [16–23]. Previously identified prognostic factors in canine primary lung neoplasia include presence of clinical signs, tumor size, degree of histopathologic differentiation, tumor grade, tumor location, and presence of lymph node metastasis [16, 17, 19, 22, 24]. A recent study demonstrated that modification of a human lung cancer staging scheme was similarly prognostic in dogs [16], providing further support for the use of canine lung tumors as a model for human lung cancer.

There are several descriptive studies that have described the imaging appearance of canine primary pulmonary tumors and emphasized an association between tumor type with lesion location [20, 23, 25]. Although these studies have resulted in revised differential lists and improved diagnostic plans, a non-invasive, objective, and reliable method needs to be developed to predict tumor type and establish prognosis in canine lung tumors prior to treatment. Radiomics refers to quantitative classification of tumor subtypes by evaluating large amounts of imaging features that are extracted from diagnostic images such as computed tomography (CT) scans [26]. Radiomic analysis provides an objective, quantitative, and non-invasive evaluation of lung tumors that may provide important information for subtype classification, tumor heterogeneity, response to therapy, and outcome [5, 8, 10, 11, 27–30]. First-order radiomic features describe the individual pixel density values of a region of interest and can be efficiently extracted from routine CT scan data.

In dogs, radiomic analysis has been used to evaluate pulmonary parenchymal changes secondary to pulmonary thromboembolism but has not yet been utilized in lung cancer [31]. The primary purpose of this exploratory, analytical study was to determine if CT radiomic data, specifically tumor shape and first-order CT features, from canine primary lung tumors is associated with histopathologic characteristics or outcome following tumor removal. We hypothesized that shape and first-order CT radiomic features would correlate with histologic subtype and characteristics and overall survival. The secondary aim of this study was to evaluate potential prognostic factors in our contemporaneous population of dogs.

## Material and methods

### Case selection

The electronic medical record database of the University of Minnesota Veterinary Medical Center was searched for dogs that underwent thoracic mass removal between January 2007 and November 2018. Inclusion criteria included identification of a primary lung tumor on a preoperative CT scan, planned surgical mass removal, and a histopathological diagnosis of primary lung neoplasia. Dogs were excluded if a complete CT image set was not available for review, if more than 3 lesions were present, or if the mass was not of pulmonary origin. Data collected from medical records included signalment, clinical signs, initial diagnostic procedures, presence or absence of metastasis, initial histopathologic diagnosis and margin status, adjunctive therapy, and time to disease progression and death. Margin status was classified as complete if reported margins were greater than or equal to 5mm or if the pathologist interpreted it as complete without measurements, incomplete if reported margins were less than 1 mm or if the pathologist interpreted margins as incomplete, or narrow if the reported margins were between 1–5 mm or the pathologist interpreted margins as narrow. The presence of clinical signs was defined as any sign that could reportedly be secondary to a lung tumor, including cough, tachypnea, dyspnea, lethargy, exercise intolerance, anorexia, hyporexia, weight loss, and discomfort [16, 17, 19]. Disease-free survival (DFS) and survival outcome were calculated from the date of the surgery.

### Histopathologic review

For all dogs, paraffin-embedded tissue blocks from each lung tumor were requested from the veterinary reference diagnostic laboratory to which surgical samples were submitted. From each paraffin block, four, 4-micron sections were cut and mounted on positively charged glass slides. One section from each block was stained with Hematoxylin and Eosin (H&E) and blindly evaluated by a board-certified veterinary pathologist (D.M.S.). Tumors were assigned a second opinion histopathologic diagnosis according to current diagnostic criteria [19, 32]. All primary adenocarcinomas were subtyped (e.g., papillary, lepidic, adenosquamous, acinar, or squamous) and graded based on a previously described scheme [19]. Tumor invasion into adjacent lung tissue, vascular spaces (blood or lymphatic), and pleura was defined as present or absent. Inflammation was scored as present or absent and its morphologic nature (mononuclear, neutrophilic, or eosinophilic) and severity was semi-quantitatively as 0 (no inflammation), 1 (mild inflammation), 2 (moderate inflammation), or 3 (severe inflammation). For cases in which paraffin blocks could not be retrieved, the original diagnosis was used for subsequent analysis.

### CT review and lung tumor feature extraction

Thoracic CT images were acquired on two CT scanners, as the CT scanner was replaced during the duration of this study. From 2007- November 2011, images were obtained with a single-slice scanner (CE CT/e, GE Healthcare, Chicago, IL). Pre- and post-contrast images were acquired at a slice thickness of 3.0–5.0 mm with no overlap. From November 2011 onward, pre- and post-contrast images were obtained with a 64-slice scanner (Toshiba Aquilion 64 CFX CT, Toshiba Medical Systems, Tustin, California). Images were acquired at a slice thickness of 1.0 mm and a slice interval of 1.0 mm and reconstructed using a slice thickness of 2.0 mm and slice interval of 2.0 mm. For all scans, iodinated contrast medium was administered intravenously by bolus (770 mg of I/kg ioversol, Optiray 350, Mallinckrodt Inc, Hazelwood, MO). CT scans were interpreted by a board-certified veterinary radiologist (C.P.O.) blinded to the histologic diagnosis and outcome. CT characteristics evaluated comprised tumor diameter and volume, location, infiltrate pattern, presence or absence of calcification, cavitation, bronchial involvement, and regional lymphadenopathy.

CT datasets were retrieved from a picture archiving and communication system (PACS) server in digital imaging and communications in medicine (DICOM) format and transferred for tumor segmentation (MIM Maestro, v. 6.8.2, MIM Software Inc, Cleveland OH). The gross tumor volume (GTV) was delineated manually on axial images on a slice-by-slice basis independently on post-contrast CT scans by a single individual (H.A.). All contours were reviewed by a single boarded radiologist (C.P.O.) for accuracy and adjusted prior to CT feature extraction from the tumor volume. The longest dimension in the axial plane for each GTV was automatically generated within MIM. The GTV was contracted axially by 0.5 mm to create the final GTV for feature extraction. To correct for noise, a 5 mm^3^ region of muscle at the level of the second thoracic vertebrae was used as reference tissue to which all other contour statistics were normalized [33]. This reference tissue served as the denominator in ratios for comparison of tumor HU data. Sixteen radiomic features characterizing shape and textural characteristics were extracted for the GTV (S1 and S2 Figs).

### Statistical analyses

Statistical analysis was performed using R version 4.0.2 software [34]. All statistical analysis was performed by a single statistician (A.R.). For continuous variables that were grouped into terciles, differences were evaluated using Kruskal-Wallis test or Wilcoxon rank-sum and Bonferroni-Holm adjustment. For continuous variables not split into terciles (MI and histologic score), Cox proportional hazards was used. For categorical variables, differences were evaluated using Fisher’s exact test. To evaluate associations with binary variables, a Wilcoxon rank-sum test was used. For correlations between radiomic features and histologic or radiologic assessment, Pearson’s product moment or Spearman’s correlation was used.

Outcome measures were estimated with Kaplan-Meier analyses and reported as median values with 95% confidence intervals (CIs). DFS was determined from the time of surgery to the date of reported respiratory or recurrent clinical signs and/or imaging or cytologic evidence of progression. Objective disease-free survival (ODFS) was determined in the dogs that had objective response assessment with imaging performed following surgery. Overall survival time (OST) and tumor-specific survival (TSS) were calculated from the date of surgery. Dogs were assumed to have died due to their tumor unless another disease was clearly identified. For DFS and ODFS, dogs were censored if they were free of disease at the time of the last follow-up. For OST analysis, dogs were censored if they were alive at the last follow-up. For TSS analysis, dogs were censored if they were alive at the last follow-up or died of a non-tumor related cause. DFS, OST and TSS were used as endpoints for assessment of imaging and tumor variables. When comparing outcomes, differences in curves was tested using the log-rank test. Measurements of tumor size were divided into terciles for evaluation of impact on outcome. Patients were censored if they were alive and/or had no disease progression noted at the time of data collection. When evaluating outcome based on imaging features, only dogs with one tumor were included in analysis. First-order features were divided into terciles and differences in survival between terciles were evaluated using the log-rank test. A p value <0.05 was considered statistically significant.

## Results

### Clinical and radiologic characteristics

A total of 65 dogs met the inclusion criteria. Dogs had a median age of 10.7 years (range 5.9–17.2 years) and a median weight of 21.7 kg (range 3.3–57.6 kg). Two dogs were diagnosed with two separate tumors, resulting in 67 tumors available for evaluation. Clinical characteristics are summarized in Table 1. Fifty-one dogs (78.5%) were treated with surgery alone, while 14 dogs (21.5%) received adjuvant systemic therapy. Systemic treatment was variable and selected at the discretion of the attending oncologist. Drugs prescribed included vinorelbine, carboplatin, doxorubicin, lomustine, 5-fluorouracil, chlorambucil, cyclophosphamide, and toceranib. Fine needle aspiration of the primary lung mass was performed prior to surgical resection for 31 (48%) tumors. Cytologic interpretation was largely concordant with the histologic classification of adenocarcinoma or non-adenocarcinoma in 25/31 (81%) tumors (Table 1). Lymph nodes were removed and submitted for histologic review in 18 dogs.

**Table 1:** Clinical characteristics of 65 dogs included for evaluation of 67 primary lung tumor radiomic and histologic features.

Characteristic	Number of dogs	Percentage
**Sex** (N = 65)		
Female spayed	34	52.3%
Male neutered	30	46.2%
Female intact	1	1.5%
**Breed** (N = 65)		
Labrador Retriever	10	15.4%
Golden Retriever	4	6.2%
Cocker Spaniel	3	4.6%
German Shepherd	3	4.6%
Mixed breed	3	4.6%
Other^a^	42	64.6%
**Presence of clinical signs at diagnosis** (N = 65)	50	77%
**Preoperative fine needle aspiration cytology** (N = 67)	31	46%
Consistent with histopathologic diagnosis	17	55%
Suggestive of histopathologic diagnosis	8	26%
Inflammation or necrosis	4	13%
Non-diagnostic	2	6%
**Histologic Diagnosis** (N = 67)		
**Adenocarcinoma** ^b^	56	84%
**Non-adenocarcinoma**	11	16%
Histiocytic sarcoma	6	9%
Sarcoma	3	4%
*Osteosarcoma*	1	1.5%
*Soft tissue sarcoma* ^c^	2	3%
Other	2	3%
*Neuroendocrine tumor*	1	1.5%
*Combined tumor* ^d^	1	1.5%
**Surgical margin status** (N = 67)		
Complete	30	44.8%
Narrow	11	16.4%
Incomplete	18	26.9%
Not reported	8	11.9%
**Presence of metastasis** (N = 65)	14	21.5%
Regional lymph node metastasis	8	12.3% (44.4%)^e^
Adenocarcinoma	9	13.8%
Non-adenocarcinoma	5	7.7%
**Adjuvant postoperative systemic therapy** (N = 65)	14	21.5%
Adenocarcinoma (N = 56)	12	21.4%
Non-adenocarcinoma (N = 11)	2	18.2%

^a^Fewer than 3 of each breed

^b^One dog with 2 adenocarcinomas

^c^One dog with 2 soft tissue sarcomas

^d^Combined carcinoma and sarcoma

^e^Percentage shown in relation to total number of dogs and in relation to regional lymph nodes that were sampled (N = 18)

All thoracic CT scans were reviewed; however, one dog with an adenocarcinoma did not have a post-contrast CT for review or for tumor segmentation and CT feature extraction. Most lung tumors were solid, mass-like lesions involving a bronchus (Table 2). Approximately 53% of tumors were located in the caudal lung lobes. Manual tumor measurements were determined by the radiologist, with a median volume of 38.3 cm^3^ (0.15–1237.20 cm^3^) and a median longest diameter in an axial plane of 4.6 cm (range 1.20–16.10 cm).

**Table 2:** Lung tumor characteristics identified on review of thoracic CT images.

CT Appearance (N = 67)	Number of tumors	Percentage
**Lesion appearance**		
Mass-like	58	87%
Pulmonary infiltrate	3	4%
Mass and infiltrate	6	9%
**Margin delineation**		
Well delineated	60	90%
Poorly delineated	7	10%
**Margin contour**		
Smooth	47	70%
Spiculated	20	30%
**Location**		
Right cranial	8	12%
Right middle	5	7.4%
Right caudal	17	25.4%
Accessory	7	10.4%
Left cranial	10	15%
Left caudal	19	28.3%
Cranial mediastinum^a^	1	1.5%
**Solid texture**	67	100%
**Presence of calcification**	21	31%
**Presence of cavitation**	17	25%
**Bubbly appearance (pseudocavitation)**	13	19%
**Bronchus involvement**		
Obstructed by tumor	7	10.4%
Penetrating tumor	27	40.3%
Peripheral, compressed by tumor	23	34.3%
No involvement	10	15%
**Presence of lymphadenopathy**	23	34%

^a^The initial diagnostic interpretation suggested cranial mediastinal origin, however this mass was confirmed to be a pulmonary adenocarcinoma on histopathology.

### Extracted radiomic data

**Fig 1:**
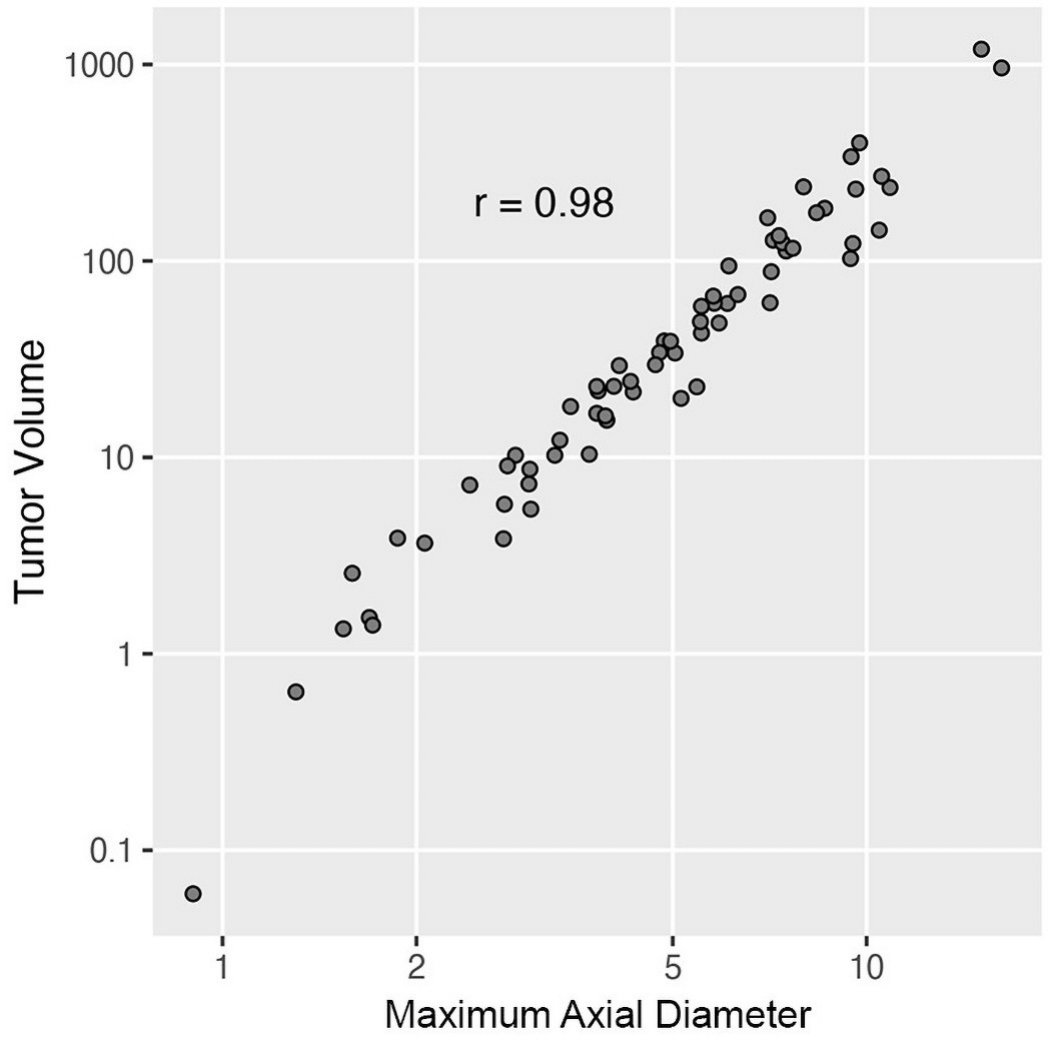
Tumor volume and axial longest diameter in canine lung tumors. The longest diameter in an axial plane and volume were automatically generated for 67 lung tumors in 65 dogs. Data are shown as individual points, with strong correlation between longest diameter and volume as determined by Pearson’s product moment correlation.

There was wide variation in first-order statistics (S2 Fig) and several features strongly correlated with each other (S1 Table). In addition to shape and texture data, tumor measurements were automatically generated from segmented tumor contours. The tumor volume was widely variable with a median volume of 34 cm^3^ (range 0.1–1196.2 cm^3^). The median longest diameter was 5.0 cm (range 0.9–16.2 cm). Tumor volume and longest axial diameter strongly correlated (*r* = 0.98) (Fig 1, S1 Table). Automatically generated tumor volumes and longest axial diameters strongly correlated with the radiologist-determined tumor volumes and longest diameters (*r* = 0.99 and *r* = 0.98, respectively).

### Histopathology

**Fig 2:**
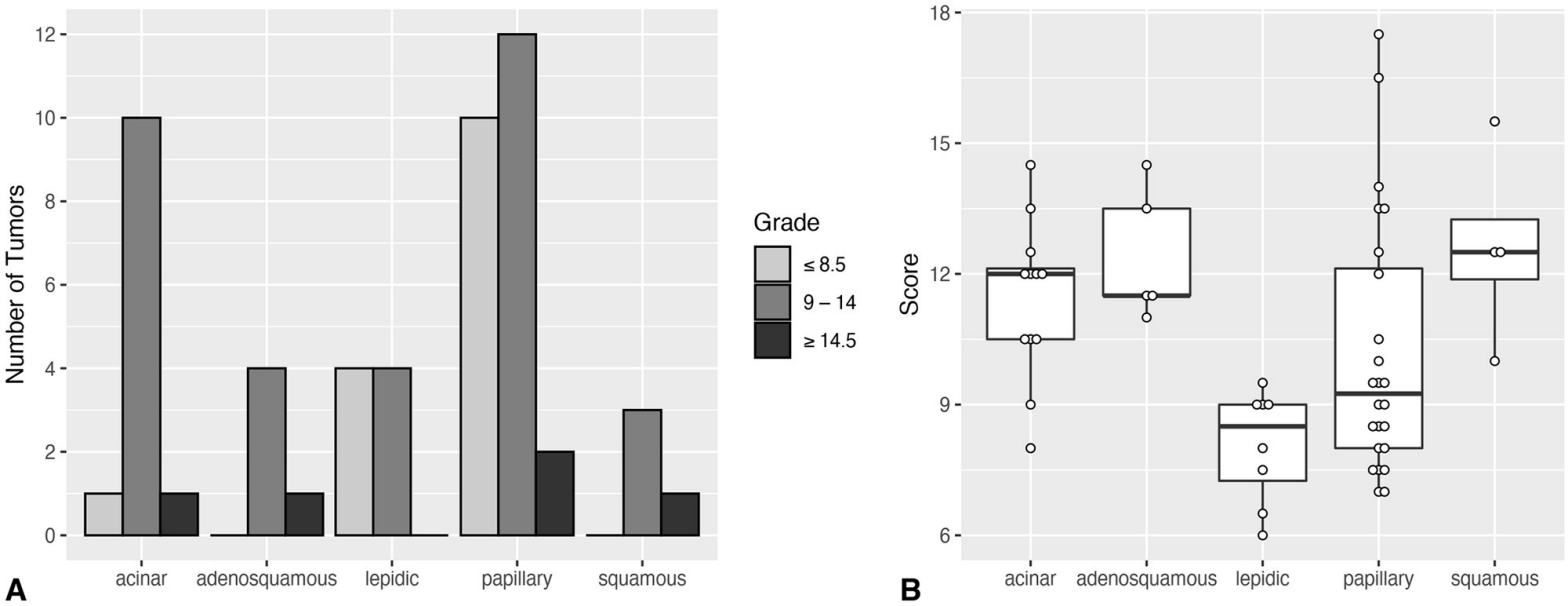
Grade and histologic score in 53 primary canine lung adenocarcinomas. (A) Total distribution of tumor grade across histologic subtype of adenocarcinoma. The number of tumors assigned to each grade is represented on the y axis, while the adenocarcinoma subtype is shown along the x axis. Grade 1 is represented by a total value of ≦ 8.5, grade 2 is represented by a total value between 9–14, and grade 3 is represented by a total score ≧ 14.5. There were no significant differences in grade between tumor types. (B) Box and whisker plot showing the histologic score, a component of grade, for canine lung adenocarcinomas. Data is presented as the median in bold with the interquartile range. Each point represents a tumor score.

Sixty-three paraffin embedded tumor blocks were acquired for histopathologic review. There was 100% agreement between the second opinion diagnosis and the original diagnosis for all cases of adenocarcinoma (N = 53) and non-adenocarcinoma tumor (N = 10). One dog had two distinct pulmonary adenocarcinomas and one dog had two distinct sarcomas. Four tissue blocks were not retrieved, representing adenocarcinomas (N = 3) and histiocytic sarcoma (N = 1); therefore, the initial diagnosis was used for subsequent statistical analyses. Of the 53 adenocarcinomas, papillary subtype and intermediate grade were most frequent (Table 3). Inflammation was present in all tumors and invasion was common (73.5%). Grade was not significantly different between adenocarcinoma subtypes (p = 0.116) (Fig 2A). However, the histologic score, a component of tumor grade, was significantly different between tumor subtypes (p = 0.003). Lepidic tumors had lower scores compared to adenosquamous (p = 0.036) and acinar subtypes (p = 0.016) (Fig 2B). There was no significant correlation between histologic subtype and local invasion. CT features did not strongly correlate to any histologic features, although several weak correlations were identified (S2 Table).

**Table 3:** Histopathology of 53 canine lung adenocarcinomas.

Characteristic (N = 53)	Number of tumors	Percentage
**Histologic subtype**		
Papillary^a^	24	45.3%
Acinar	12	22.6%
Lepidic	8	15.1%
Adenosquamous	5	9.4%
Squamous	4	7.6%
**Grade**		
1	15	28.3%
2^a^	33	62.3%
3	5	9.4%
**Presence of invasion** ^a^	39	73.5%
**Presence of inflammation**	53	100%

^a^One dog had 2 distinct papillary, grade 2 adenocarcinomas with evidence of invasion.

### Outcome

#### All dogs with primary lung tumors

**Fig 3:**
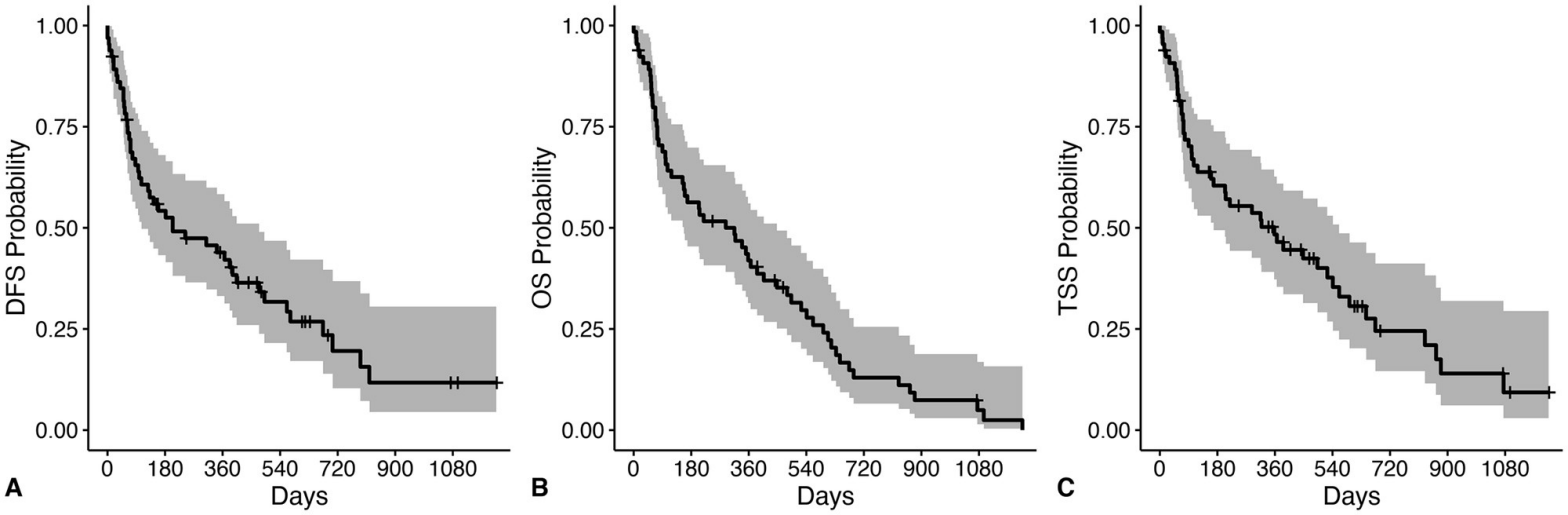
Outcome for all dogs with primary lung tumors following surgical resection. Disease-free survival (DFS), overall survival (OS), and tumor-specific survival (TSS) curves in 65 dogs with primary lung tumors following surgery. Ticks represent dogs censored from analysis and shaded regions represent the 95% confidence interval (CI). Dogs were censored from DFS if they were free of disease at the time of last follow-up. Dogs were censored from TSS if they were alive at the time of last follow-up or died due to a known cause unrelated to a lung tumor. (A) The median DFS was 204 days (CI: 106–474 days). (B) The median OST was 315 days (CI: 157–448 days). (C) The median TSS was 358 days (CI: 168–561 days).

**Fig 4:**
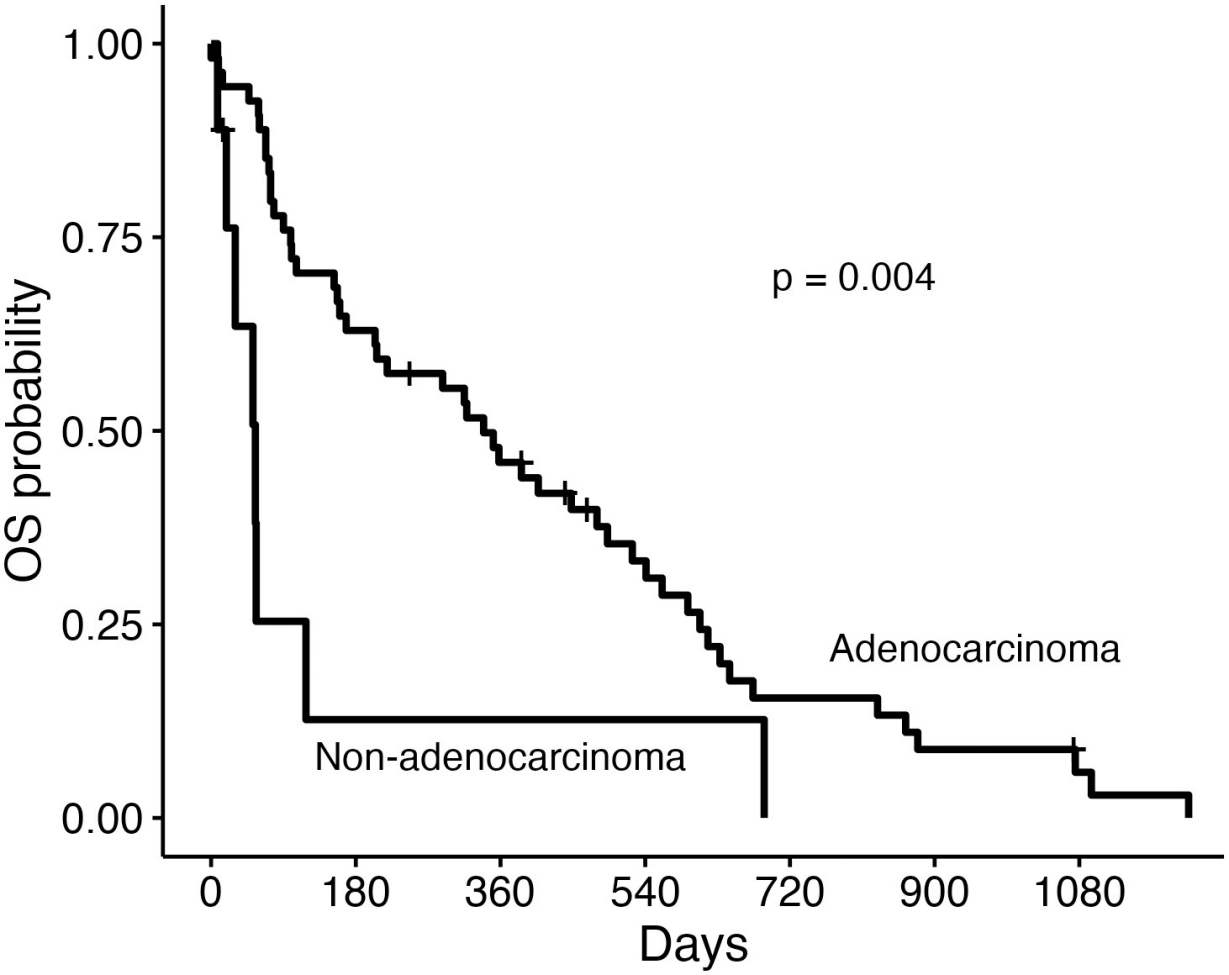
Overall median survival time for dogs with primary lung adenocarcinoma or non-adenocarcinoma following surgical resection. Overall survival (OS) curves for 65 dogs based on broad histologic classification of adenocarcinoma or non-adenocarcinoma. Ticks represent dogs censored from analysis. Dogs were censored if they were alive at the time of last follow-up. The median overall survival time (OST) for dogs with adenocarcinoma was 339 days (95% confidence interval [CI]: 204–524 days) while the median OST for dogs with tumors other than adenocarcinoma was 55 days (CI: 30-not reached).

For all 65 dogs, the median DFS, OST and TSS was 204 days, 315 days, and 358 days, respectively (Fig 3). For the 45 dogs with objective response assessment available, the ODFS was 382 days. Dogs with adenocarcinoma survived significantly longer than dogs with non-adenocarcinoma (OST 339 days versus OST 55 days, p = 0.004) (Fig 4). The presence of metastasis negatively impacted median DFS (391 days versus 25 days, p<0.001), median OST (366 days versus 56 days, p<0.001) and median TTS (493 days versus 56 days, p<0.001). Chemotherapy or toceranib treatment did not significantly impact outcome measures.

Smaller tumors were significantly (p = 0.013) more likely than larger tumors to be completely excised. Completely excised tumors had a median volume of 19.8 cm^3^, whereas narrowly and incompletely excised tumors had a median volume of 57.2 cm^3^ and 60 cm^3^, respectively. Regardless of tumor histology, longest axial tumor diameter and volume were significantly associated with all outcome measures (Table 4).

**Table 4:** Outcome parameters for dogs with lung tumors based on maximum tumor diameter and volume.

	DFS (days)		OST (days)		TSS (days)	
**Maximum axial tumor diameter (cm)**						
0.90–3.81	674	p<0.001	608	p<0.001	674	p<0.001
3.82–6.08	404		493		524	
6.09–16.20	63		72		74	
**Tumor Volume (cm** ^**3**^ **)**						
0.06–17.70	674	p<0.001	618	p<0.001	674	p<0.001
17.71–66.60	404		480		524	
66.61–1196.20	61		73		73	

Mean HU ratio (p = 0.042) and median mean HU ratio (p = 0.042) were significantly higher in adenocarcinomas compared to non-adenocarcinomas (S2 Fig). Regardless of tumor type, higher values for several radiomic features, including integral total (HU/ml), max mean HU ratio, and total HU, were associated with shorter DFS, OST and TSS (Table 5).

**Table 5:** CT features associated with outcome measures in dogs with lung tumors.

CT Feature	DFS	OST	TSS
Integral total (HU/ml)	p<0.001	p<0.001	p<0.001
Max mean HU ratio	p = 0.039	p = 0.005	p = 0.018
Total (HU)	p<0.001	p<0.001	p<0.001

#### Dogs with primary lung adenocarcinoma

**Fig 5:**
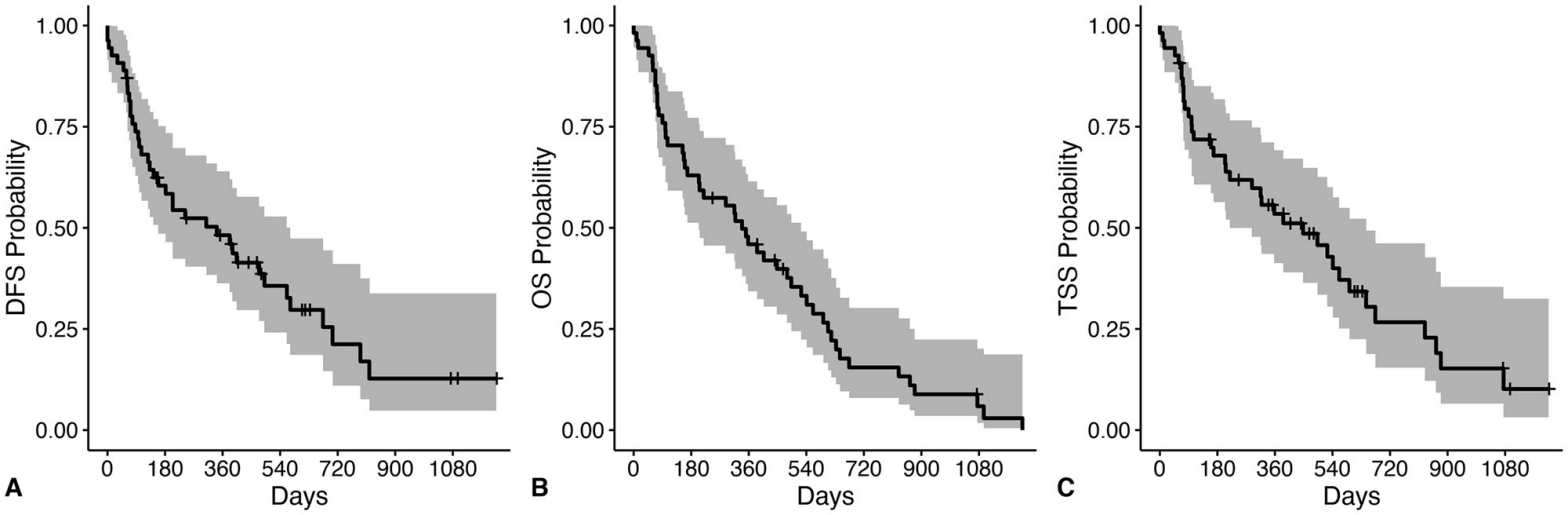
Outcome for dogs with primary lung adenocarcinoma following surgical resection. Disease-free survival (DFS), overall survival (OS), and tumor-specific survival (TSS) curves for 55 dogs with lung adenocarcinoma following surgery. Ticks represent dogs censored from analysis and shaded regions represent the 95% confidence interval (CI). Dogs were censored from DFS if they were free of disease at the time of last follow-up. Dogs were censored from TSS if they were alive at the time of last follow-up or died due to a known cause unrelated to a lung tumor. (A) The median DFS was 342 days (CI: 159–572 days). (B) The median overall survival time (OST) was 339 days (CI: 204–524 days). (C) The median TSS was 448 days (CI: 219–645 days).

**Fig 6:**
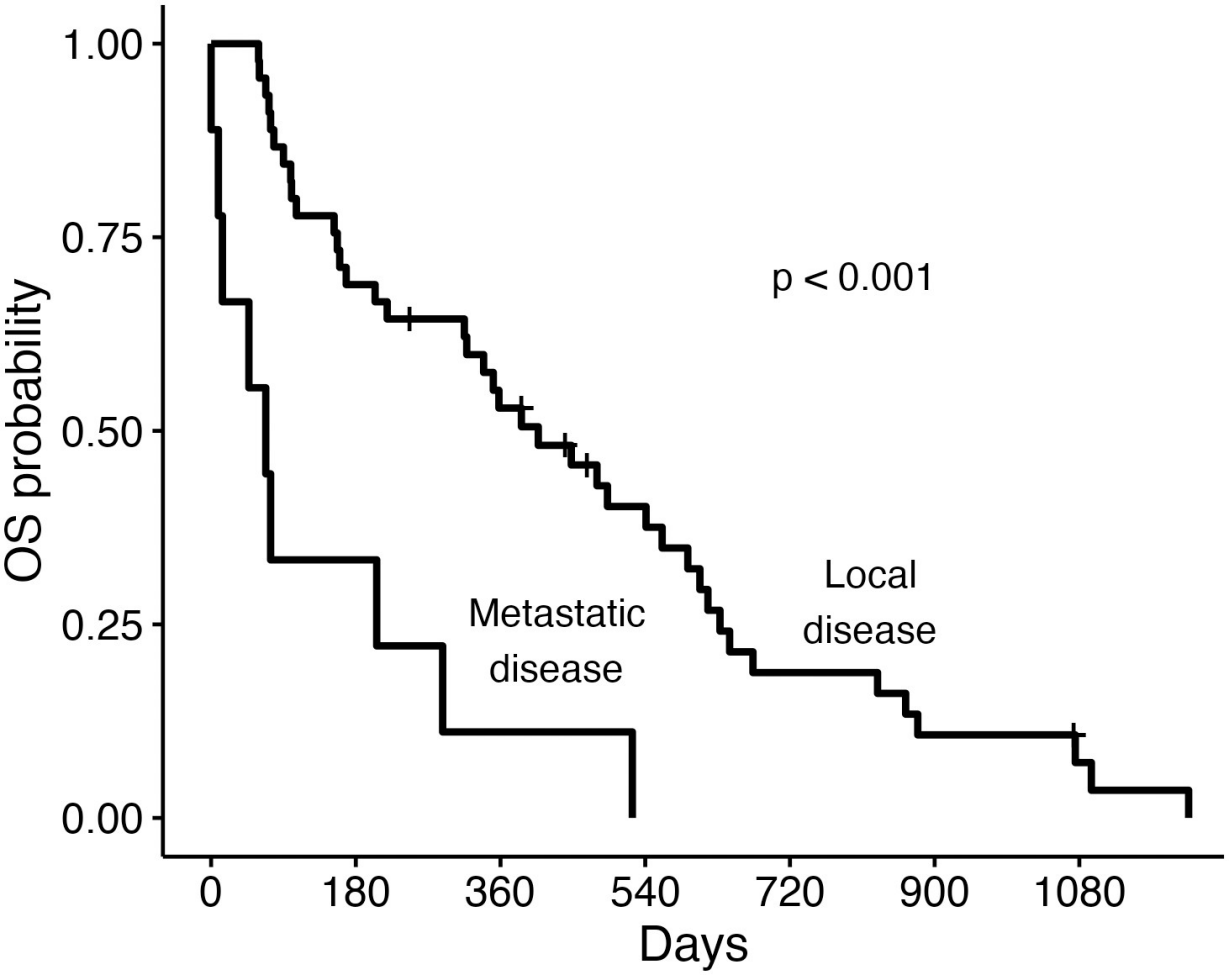
Overall median survival time for dogs with adenocarcinoma with and without metastasis. Overall survival (OS) curves for dogs with lung adenocarcinoma that presented with (N = 9 dogs) or without metastasis (N = 45) at the time of diagnosis. Ticks represent dogs censored from analysis. Dogs were censored if they were alive at the time of last follow-up. The median overall survival time (OST) for dogs with adenocarcinoma that presented with local disease was 407 days (95% confidence interval [CI]: 315–608 days) while the median OST for dogs with metastasis was 68 days (CI: 14-not found).

For the 55 dogs with lung adenocarcinoma, the median DFS, OST and TTS were 342 days, 339 days and 448 days, respectively (Fig 5). For the 39 dogs with objective response assessment available, the median ODFS was 403 days. Dogs without metastatic disease had a median OST of 407 days, which was significantly (p<0.001) longer than the median OST of 68 days for the 9 dogs with metastasis (Fig 6). Similarly, the median TSS for dogs without metastasis was 541 days compared to 68 days (p<0.001) for dogs with metastasis. Dogs (N = 12) that received adjuvant systemic therapy did not have improved outcome (median OST 320 days) compared to dogs (N = 42) that did not receive systemic chemotherapy or toceranib (median OST 339 days) following surgery (p = 0.83). No significant associations were present between DFS, OST, or TTS and subtype, grade, presence of tumor invasion, or MI score (Table 6).

**Table 6:** Outcome for dogs with lung adenocarcinoma following surgical resection.

Histologic characteristic	DFS (days)		OST (days)		TSS (days)	
**Subtype**						
Acinar	382	p = 0.98	386	p = 0.49	448	p = 0.81
Adenosquamous	404		90		541	
Lepidic	435		450		493	
Papillary	204		345		593	
Squamous	408		480		480	
**Grade**						
1	574	p = 0.15	407	p = 0.18	674	p = 0.17
2	342		358		493	
3	72		339		386	
**Invasion**						
Absent	256	p = 0.95	313	p = 0.46	334	p = 0.67
Present	404		358		541	
**MI Score**						
1	474	p = 0.21	480	p = 0.45	561	p = 0.25
2	204		204		358	
3	362		369		386	
4	138		279		432	

**Fig 7:**
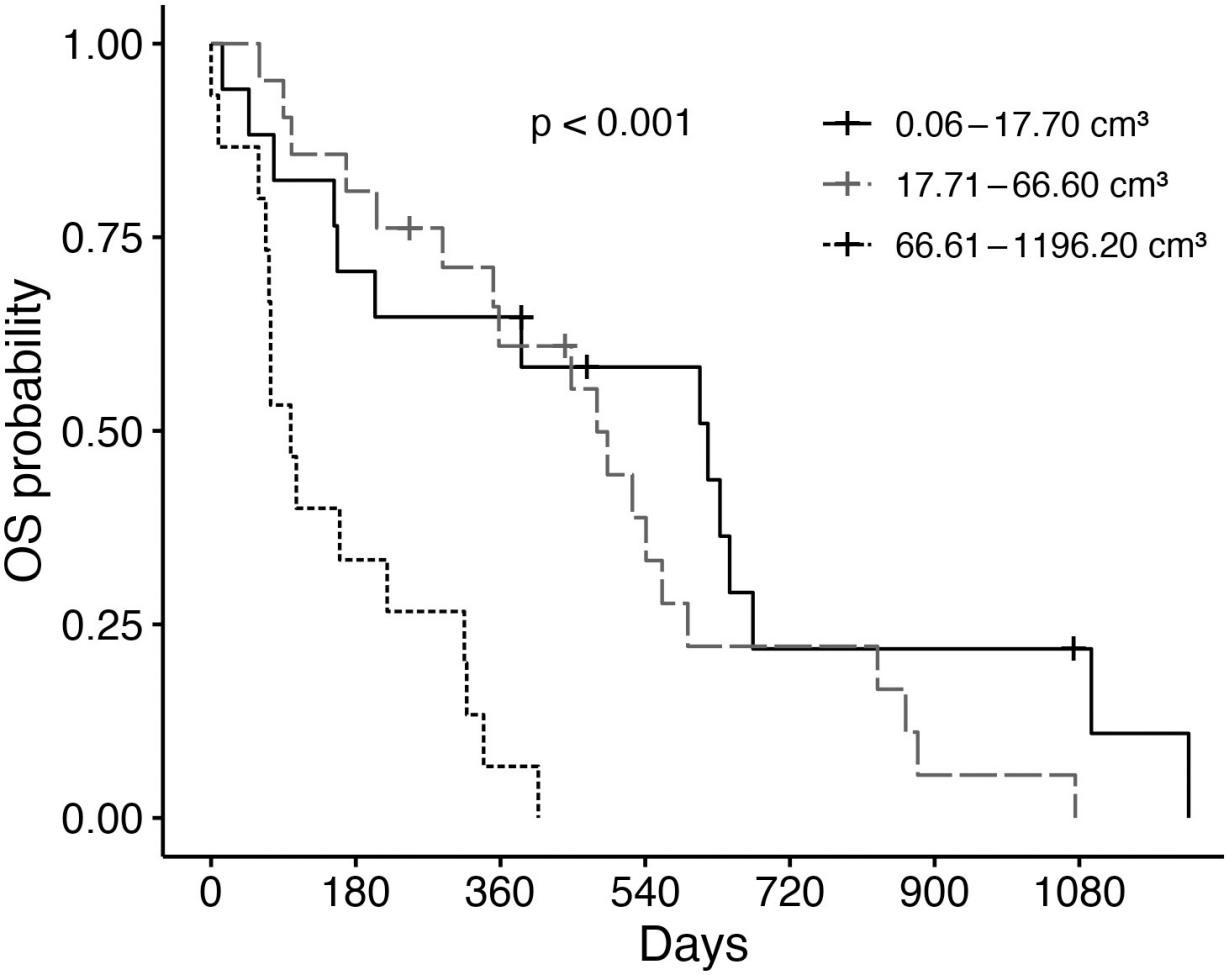
Overall median survival time for dogs with adenocarcinoma stratified by volume. Overall survival (OS) curves for dogs with lung adenocarcinoma were stratified by tumor volume. Ticks represent dogs censored from analysis. Dogs were censored if they were alive at the time of last follow-up. The median overall survival time (OST) for dogs with smaller (0.06–17.70 cm^3^) lung adenocarcinomas was 618 days (95% confidence interval [CI]: 204 days-not found) compared to 480 days for dogs with intermediate (17.71–66.60 cm^3^; CI: 351–829 days) tumors and 99 days for dogs with large (66.61–1196.20 cm^3^; CI: 72–318 days) tumors.

Dogs with smaller tumors, as measured by the maximum tumor diameter or volume, had significantly improved outcome measures compared to dogs with larger tumors (Table 7, Fig 7). Incompletely excised tumors had significantly shorter DFS, OST, and TSS compared to tumors narrowly or completely excised (Table 8). Larger tumor volume was also associated with the presence of histologic invasion to adjacent tissue (p = 0.044). Among the 55 dogs with adenocarcinomas, there was no association with tumor volume and subtype (p = 0.758).

**Table 7:** Outcome parameters for dogs with lung adenocarcinoma based on tumor diameter and volume.

	DFS (days)		OST (days)		TSS (days)	
**Maximum axial tumor diameter (cm)**						
0.90–3.81	674	p<0.001	608	p<0.001	674	p<0.001
3.82–6.08	404		493		541	
6.09–16.20	72		87		99	
**Tumor Volume (cm** ^**3**^ **)**						
0.06–17.70	674	p<0.001	618	p<0.001	674	p<0.001
17.71–66.60	404		480		524	
66.61–1196.20	72		99		99	

**Table 8:** Outcome parameters for dogs with lung adenocarcinoma based on surgical margin status.

Surgical margins	DFS (days)		OST (days)		TSS (days)	
Complete	491	p<0.001	541	p<0.001	593	p<0.001
Narrow	819		524		524	
Incomplete	87		134		168	

Increasing mitotic index significantly decreased DFS (HR 1.03; CI: 1.009–1.060; p = 0.008). Increasing total score also significantly decreased DFS (HR 1.14; CI: 1.005–1.290; p = 0.042). Other histologic features were not significantly associated with outcome.

Similar to the analysis for all dogs, increased values for some first-order CT features were significantly associated with shorter DFS, OST and TSS and increased max HU to mean HU ratio was associated with shorter OST and TSS (Table 9). Three of these features—integral total (HU/ml), integral total mean HU ratio, and total HU were strongly positively correlated to each other and to both longest tumor diameter and volume (S1 Table).

**Table 9:** Analysis of CT features associated with outcome measures in dogs with lung adenocarcinoma.

CT Feature	DFS p value	OST p value	TSS p value
Integral total (HU/ml)	<0.001*	<0.001*	<0.001*
Integral total mean HU ratio	<0.001*	<0.001*	<0.001*
Kurtosis	0.39	0.64	0.67
Max (HU)	0.29	0.11	0.19
Max mean HU ratio	0.063	0.008*	0.031*
Mean (HU)	0.81	1.00	0.97
Mean HU ratio	0.31	0.58	0.20
Median (HU)	0.85	0.95	0.94
Median mean HU ratio	0.43	0.56	0.24
Min (HU)	0.50	0.16	0.31
Min mean HU ratio	0.64	0.21	0.26
Skewness	0.70	0.52	0.77
Standard deviation	0.39	0.79	0.54
Standard deviation mean HU ratio	0.70	0.89	0.65
Total (HU)	0.004*	0.002*	0.002*

## Discussion

This is the first study to evaluate CT radiomic features in canine lung tumors. Several shape and first-order CT features were associated with tumor type, histologic invasion and outcome. Importantly, two distinct features, mean HU ratio and median mean HU ratio, were significantly different between pulmonary adenocarcinomas and non-adenocarcinomas. Results support that further investigation to determine how texture and shape analysis can provide prognostic ability, such as subtype, is warranted. The ability to differentiate pulmonary adenocarcinoma from a non-adenocarcinoma is clinically relevant, as outcome following surgery was significantly better for dogs with adenocarcinoma. Use of radiomic analysis for this purpose could therefore improve presurgical prognostic information, such as better identifying dogs that may benefit from further diagnostics. This is an important first step towards better patient stratification in veterinary oncology, with an aim to inform treatment decisions that improve outcome.

First-order, or pixel intensity, CT features evaluated here provided only weak correlations to several histologic features. Interestingly, none of the histologic variables, aside from tumor type, were associated with prognosis, while four CT features and tumor volume were significantly associated with DFS, OST and TSS. Three of the CT features, namely integral total (HU/ml), integral total mean HU ratio, and total HU were strongly correlated to volume; thus, tumor volume may indeed be the most important prognostic feature. However, max mean ratio was not strongly associated with the other CT intensity features or with volume, supporting that evaluating CT pixel data holds promise as a potentially clinically useful indicator. Although max mean ratio was not significantly related to histopathologic features evaluated in this study, it may provide other pertinent information reflective of intratumoral heterogeneity and biologic behavior. Tumor heterogeneity is a well-established marker of tumor behavior and has been associated with poor prognosis in human pulmonary carcinoma [30, 35, 36]. In this pilot study, we sought to identify efficient evaluation of first-order radiomic features, as no postprocessing or transfer of images out of the contouring software was necessary and values were automatically provided. Further investigation is warranted to evaluate higher order features that evaluate pixel intensity and their spatial relationships to each other.

Tumor size has consistently documented to be a useful prognostic factor in canine primary lung neoplasia [16, 22, 24]. Results here support that tumor burden is important, as dogs with smaller tumors had significantly improved outcomes. Results also demonstrated that multiple methods can be used by which to assess tumor burden that are associated with outcome, which carries clinical relevance. Volumetric tumor measurements from segmented volumes were considered most accurate and were used for our statistical analyses. However, contouring of tumors can be time-consuming and requires additional software [37]. There was very strong correlation between contoured volumes and maximum axial diameter and those measurements obtained from the radiologist’s assessment. This result is contrary to a recent study that showed poor consistency between manual volume or maximum diameter measurements determined by a radiologist and automatically generated measurements from segmented canine nasal tumor contours [37]. In that study, manual radiologist-generated measurements significantly underestimated tumor volume and maximum diameter compared to those obtained following segmentation [37]. One possible explanation for this difference is nasal tumors are irregular in shape and can be difficult to differentiate from surrounding nasal secretions and inflammation in comparison to lung tumors, which tend to be well delineated (Table 2).

While only weak correlations were noted between first-order features and histology, further investigation is warranted into other histologic features including expression of immunohistochemical targets, such as epidermal growth factor receptor (EGFR), that improve personalized therapy. Notably, activating mutations of EGFR in human lung carcinoma are a favorable predictor of response to tyrosine kinase inhibitors, and the standard first line therapy may be altered with specific mutations [38, 39]. First-order and higher-order CT radiomic features have been shown to predict EGFR status in human lung tumors [27, 40, 41], and the incorporation of these features into the diagnostic paradigm may be beneficial for early treatment stratification. Although further work needs to be done to clarify the role of EGFR in canine lung tumors, one study demonstrated that canine lung tumors with high EGFR expression by immunohistochemistry showed shorter survival compared to dogs with low EGFR expression, although this difference was not significant [42]. Future prospective evaluations of radiomic features in canine lung tumors would optimally include serial banking of lung tumor tissue at diagnosis, response (if not excised), and recurrence to allow for more detailed histological sectioning, RNA sequencing, immunohistochemistry, and other molecular testing as data is produced. Large volumes of imaging and histologic data will be needed to provide stronger correlative data.

Accurate identification of markers that predict response could provide valuable information about treatment options and utilization of systemic therapy when surgical intervention is not possible or elected. A recent study reported that metronomic therapy treatment resulted in improved survival compared to maximally tolerated chemotherapy for primary pulmonary adenocarcinomas [43]. With further evaluation, it may be possible to identify CT features that could identify dogs more likely to respond to chemotherapy, targeted therapy or radiation therapy. For example, intratumoral and peritumoral CT texture and shape features in one study were associated with pathologic response following chemoradiation in patients with advanced non-small cell lung cancer [44]. In our study, while grade was not different among tumor subtypes, lepidic tumors were more likely to have a lower histologic score compared to acinar and adenosquamous tumors. This discrepancy may be due to histologic score being a continuous measure, rather than a categorical measure like grade. Although first-order CT features were not strongly associated with subtype, histologic score or grade in this study, it is possible that evaluation of a larger numbers of tumors and/or the incorporation of higher order feature analysis may better identify radiomic features better correlated to histologic features of aggressive behavior.

Our results also supported that a histologic diagnosis of non-adenocarcinoma and presence of metastasis at diagnosis, both previously identified prognostic factors, significantly affected outcome following surgery. Adjuvant chemotherapy did not improve outcome in this population of dogs, which is consistent with a previous study [16]. It is possible that clinicians elected to prescribe adjuvant chemotherapy to dogs perceived to have more aggressive tumors, thus introducing bias. The optimal chemotherapy drug and protocol for canine lung tumors has not been defined, leading to clinician discretion and optimizing the choice and dosage of chemotherapy may lead to different results. It is also possible that our finding represents a type II error given that only a minority of dogs with adenocarcinoma (18%) received chemotherapy. Interestingly, results of our study with a median OST of 318 days for dogs that received surgery alone is similar to a recent study that reported a median OST of 324 days for dogs that underwent surgery and adjuvant vinorelbine for primary pulmonary carcinoma [45], supporting that work needs to be done to identify the role of systemic therapy for canine lung tumors. Completeness of tumor excision was also a positive prognostic factor. Completely excised tumors were significantly smaller compared to those narrowly or incompletely excised, thus highlighting the importance of early detection and intervention.

One of the primary strengths of this study is that the methodology can be easily translated to other species. Second opinion histopathology for lung carcinomas using a previously published grading scheme [19] was also performed to strengthen the relationships found with CT radiomic analysis. Additional grading was not performed in the population of dogs without adenocarcinoma given the small number of dogs with specific histologic subtypes. The small numbers of dogs with non-adenocarcinoma also prevented subgroup evaluation of first-order features and outcome. In this particular study, all dogs had surgical intervention, thus results may not be attributable to dogs with advanced disease not amenable to surgery. Additional study should be made in dogs with measurable disease.

There were some limitations to this study, namely associated with the group size and the lack of standardization of imaging and treatment protocols for dogs with lung tumors over the study period. Importantly, the use of two CT units over the course of this study in conjunction with the lack of standard image acquisition is a limitation. We included dogs that were imaged on both scanners as neither scanner was associated with a standardized lung tumor imaging protocol and tumor sections were available for review, maximizing case numbers. This study was not adequately powered to evaluate for differences between the two scanners as the majority of dogs were imaged with one machine (Toshiba). Many features extracted evaluate x-ray attenuation (via measurement of HUs) within the tumor and the accuracy of CT attenuation on each machine was assessed as part of routine quality assurance procedures. However, it is possible that small variances in CT attenuation between CT units slightly alters results, since differences between units have not been fully studied or characterized [46].

It is also important to recognize that dogs with less aggressive disease may have been selected for, given that all dogs had their lung tumors removed. Additional differences may be evident with a larger population of dogs that includes those that did not receive definitive treatment. Of potential importance, not all dogs had regional lymph nodes extirpated and evaluated in this study thus the presence of metastasis may have been underreported, as 23 dogs had lymphadenopathy noted on CT, but only 18 cases had lymph node sampling performed. The presence of lymph node metastasis has been reported to negatively influence survival [21]. A recent report supported this finding, as dogs without lymph node metastasis had longer survival than dogs without metastasis, although the difference was not statistically significant [17]. Finally, not all dogs underwent objective imaging following surgery, which may have altered the DFS assessment. Due to statistical concerns about bias if dogs without objective imaging were excluded, the use of DFS for all 65 dogs was deemed more appropriate for subsequent analysis. All dogs with recurrent clinical signs had respiratory signs in DFS analysis, except for 1 dog. This dog had recurrence of vomiting and lethargy, which were identical to the clinical signs at the dog’s diagnosis of primary pulmonary histiocytic sarcoma.

Future studies that seek to pursue radiomic analysis for specific tumors should standardize the workflow, both within and between institutions. As previously mentioned, it is ideal to utilize the same CT and imaging protocol, but additional efforts to standardize a radiomics workflow are necessary. Standards need to be set that include timing of contrast injection (if used), respiratory protocols (if dogs are ventilated), postprocessing techniques, segmentation of regions of interest, feature extraction software, and reporting of radiomic data [26, 35, 46]. The lack of standard workflow introduces potential variations in radiomic data, limiting the generalizability of radiomic studies across various institutions. However, for robust statistical analyses, large numbers of dogs will need to be included given the large numbers of features that can be analyzed. This supports the need to establish multi-institutional efforts that standardize treatment, response, and outcome assessment.

Based on the identified correlations in this study, which evaluated rapidly extracted radiomics features using commercially available software, additional study of higher order features from post-processed images to evaluate prognostic and predictive utility is warranted. Radiomic features are collected with no additional risk to the patient and with little to no additional cost. Evaluation of first-order statistics from pre-contrast CT datasets and contours of a segment of the entire tumor volume may also provide valuable information. The ability to isolate a smaller portion of the tumor, reducing the time to collect the data, would make using radiomic analysis more feasible on a wide-spread scale. Correlation of imaging features with histologic or molecular markers associated with tumor aggressive behavior or therapeutic response would also be valuable, as findings have the potential to alter diagnostic and treatment paradigms.

## Conclusion

Quantitative analysis using first-order CT statistics may provide novel, non-invasive information about canine primary pulmonary tumors. Further investigation of radiomic feature evaluation and workflow optimization is warranted in prospective trials of dogs with standardized imaging and post-processing criteria to validate its potential prognostic utility.

## Supporting information

S1 Table Relationships between CT variables. First-order CT radiomic features. Features demonstrating significant very strong (rsp > 0.89), strong (rsp 0.70–0.89) are presented. Correlating variables are only shown once.
(DOCX)

S2 Table CT features correlating with histologic parameters. Histology and CT feature correlations. Weak (0.20–0.39) correlations are shown; there were no very strong (rsp > 0.89), strong (rsp 0.70–0.89), or moderate (0.4–0.69) correlations.
(DOCX)

S1 Fig Representation of the workflow utilized for the segmentation of lung tumors and feature extraction. The turquoise area (white arrow) represents the manually contoured tumor on post contrast CT and the yellow region (black arrow) represents the reference muscle tissue. CT features are automatically generated by MIM software for each tumor, as demonstrated, with values normalized to the reference tissue. The axial CT image is shown at a window width of 500 HU and a window level of 70 HU.
(TIF)

S2 Fig CT radiomic features from canine primary lung tumors. First order CT radiomic features were extracted. Tumors were divided into adenocarcinoma (AC) and non-adenocarcinoma (Non-AC) in each box and whisker plot to indicate the variability in values within each broad histologic category. Data are shown as the median and interquartile range. Each data point represents an individual tumor. P values represent results from Wilcoxon rank-sum test; values < 0.05 were considered significant.
(TIF)
