# Supplementary material for: Computed tomography radiomic features hold prognostic utility for canine lung tumors: An analytical study
Source: PLoS One. 2021 Aug 17;16(8):e0256139. doi: 10.1371/journal.pone.0256139 (PMC8370631; doi:10.1371/journal.pone.0256139)
Supplement: S1 Table — First-order CT radiomic features. Features demonstrating significant very strong (rsp > 0.89), strong (rsp 0.70–0.89) are presented. Correlating variables are only shown once. (DOCX) [file pone.0256139.s001.docx]

**S1 Table. Relationships between CT variables**. First-order CT radiomic features. Features demonstrating significant very strong (*r*_sp_ > 0.89), strong (*r*_sp_ 0.70-0.89) are presented. Correlating variables are only shown once.

| **CT Feature** | **CT Feature** | **Correlation estimate (*r*_sp_)** |
| --- | --- | --- |
| Longest axial diameter (cm) | Integral total (HU/ml) | 0.92 |
|  | Integral total mean HU ratio | 0.89 |
|  | Total (HU) | 0.85 |
|  | Volume (cm^3^) | 0.98 |
| Volume (cm^3^) | Integral total (HU/ml) | 0.94 |
|  | Integral total mean HU ratio | 0.92 |
|  | Total (HU) | 0.86 |
| Integral total (HU/ml) | Integral total mean HU ratio | 0.98 |
|  | Total (HU) | 0.93 |
| Total (HU) | Integral total mean HU ratio | 0.91 |
| Max (HU) | Max Mean HU ratio | 0.73 |
| Mean (HU) | Median (HU) | 0.90 |
|  | Mean HU ratio | 0.77 |
| Mean HU ratio | Median (HU) | 0.77 |
|  | Median mean HU ratio | 0.93 |
| Median (HU) | Median mean HU ratio | 0.82 |
| Min (HU) | Min mean HU ratio | 0.96 |
| Standard deviation | Standard deviation mean HU ratio | 0.76 |
