# Supplementary material for: Computed tomography radiomic features hold prognostic utility for canine lung tumors: An analytical study
Source: PLoS One. 2021 Aug 17;16(8):e0256139. doi: 10.1371/journal.pone.0256139 (PMC8370631; doi:10.1371/journal.pone.0256139)
Supplement: S2 Table — Histology and CT feature correlations. Weak (0.20–0.39) correlations are shown; there were no very strong (rsp > 0.89), strong (rsp 0.70–0.89), or moderate (0.4–0.69) correlations. (DOCX) [file pone.0256139.s002.docx]

**S2 Table. CT features correlating with histologic parameters.** Histology and CT feature correlations. Weak (0.20-0.39) correlations are shown; there were no very strong (*r*_sp_ > 0.89), strong (*r*_sp_ 0.70-0.89), or moderate (0.4-0.69) correlations.

| **Histologic characteristic** | **CT feature** | **Correlation estimate (*r*_sp_)** |
| --- | --- | --- |
| Grade | Kurtosis | 0.20 |
|  | Max mean HU ratio | 0.23 |
|  | Mean (HU) | -0.22 |
|  | Median (HU) | -0.30 |
|  | Median mean HU ratio | -0.22 |
|  | Min (HU) | -0.21 |
|  | Standard deviation | 0.26 |
|  | Standard deviation mean HU ratio | 0.23 |
| MI | Median (HU) | -0.23 |
|  | Skewness | 0.20 |
| MI score | Kurtosis | 0.20 |
|  | Median (HU) | -0.25 |
| Score | Max (HU) | 0.20 |
|  | Max mean HU ratio | 0.23 |
|  | Median (HU) | -0.24 |
|  | Standard deviation | 0.24 |
|  | Standard deviation mean HU ratio | 0.23 |
