# Supplementary figures and images for: Computed tomography radiomic features hold prognostic utility for canine lung tumors: An analytical study
Source: PLoS One. 2021 Aug 17;16(8):e0256139. doi: 10.1371/journal.pone.0256139 (PMC8370631; doi:10.1371/journal.pone.0256139)

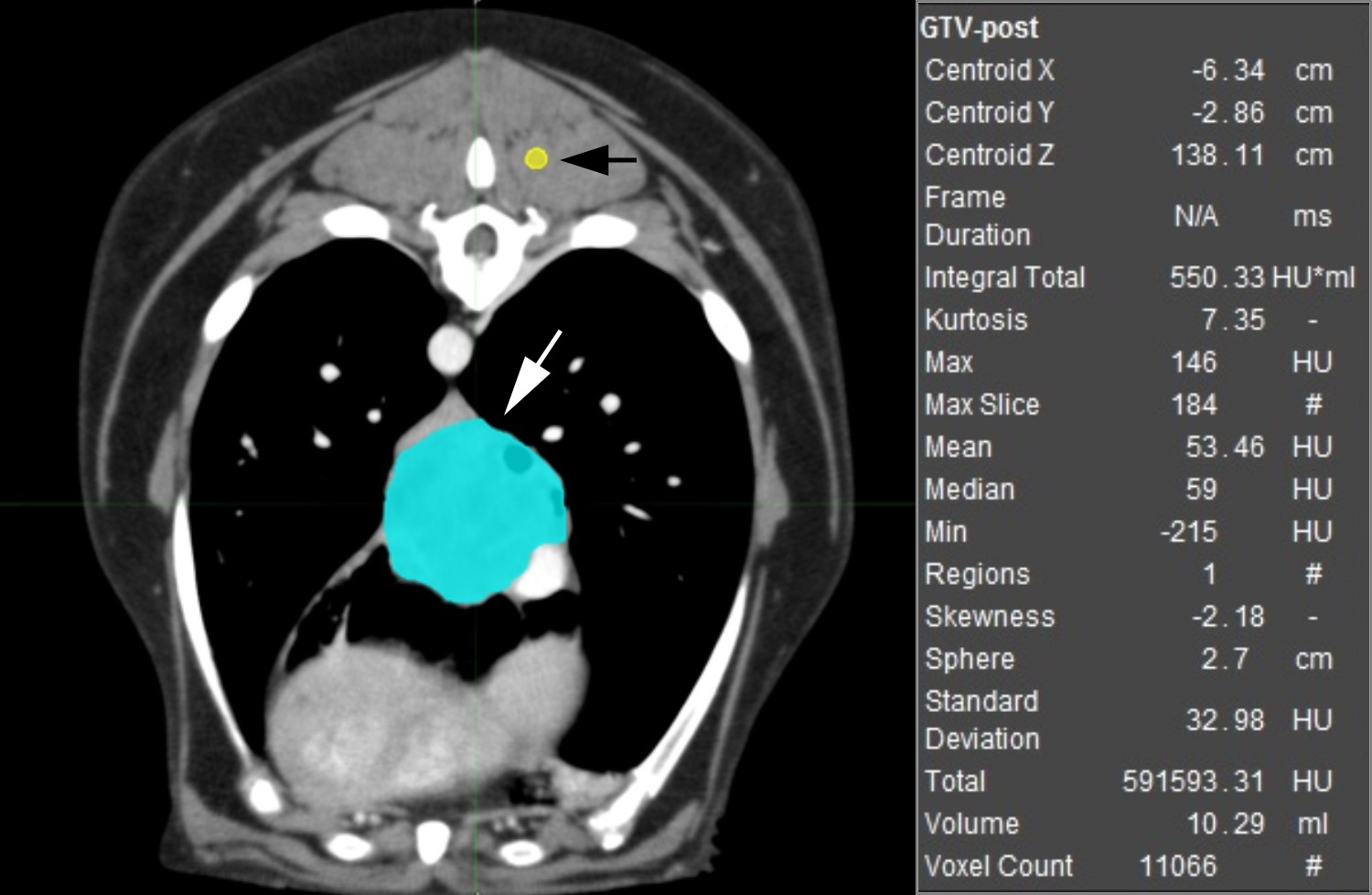

Supplement: S1 Fig — The turquoise area (white arrow) represents the manually contoured tumor on post contrast CT and the yellow region (black arrow) represents the reference muscle tissue. CT features are automatically generated by MIM software for each tumor, as demonstrated, with values normalized to the reference tissue. The axial CT image is shown at a window width of 500 HU and a window level of 70 HU. (TIF) [file pone.0256139.s003.tif]

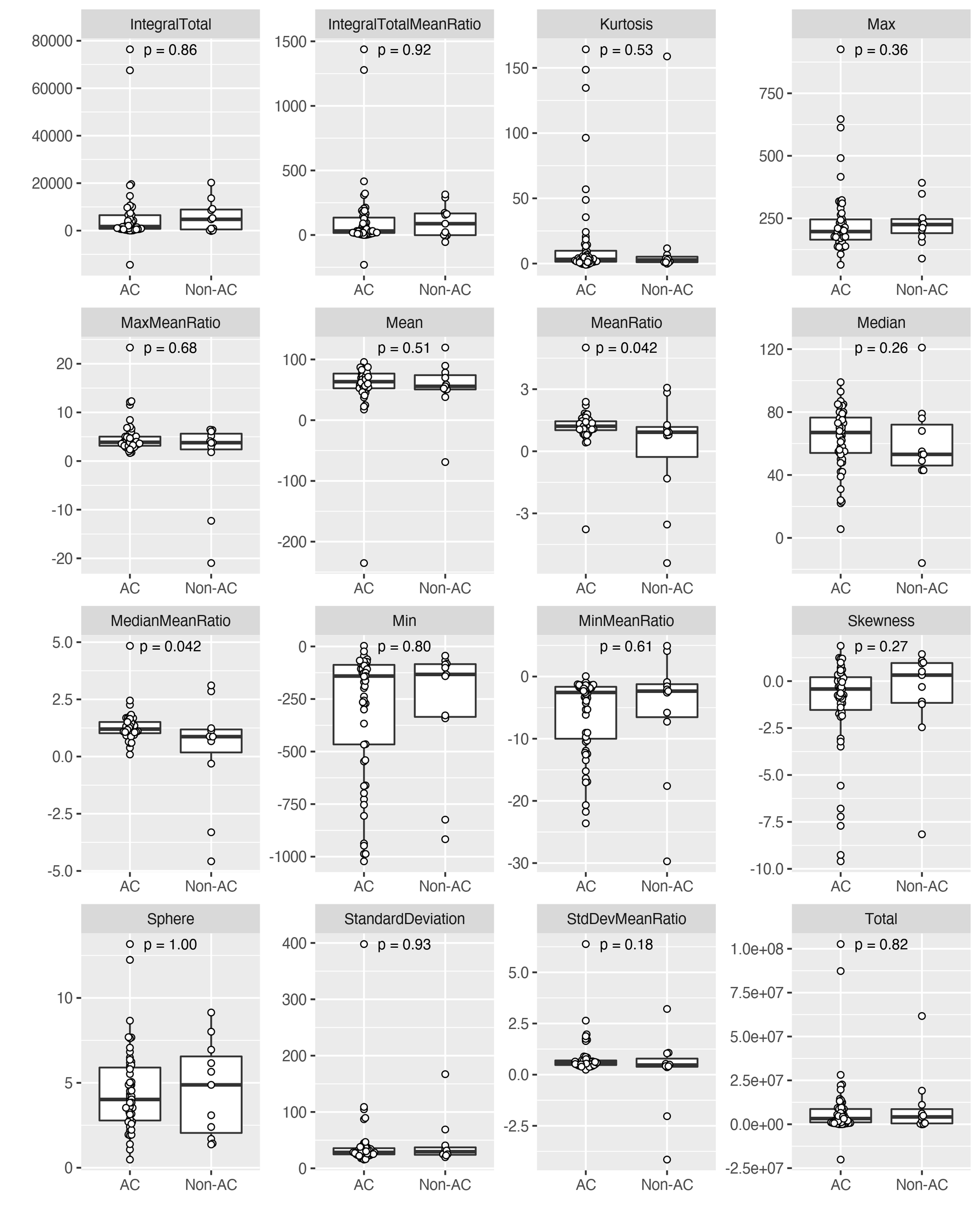

Supplement: S2 Fig — First order CT radiomic features were extracted. Tumors were divided into adenocarcinoma (AC) and non-adenocarcinoma (Non-AC) in each box and whisker plot to indicate the variability in values within each broad histologic category. Data are shown as the median and interquartile range. Each data point represents an individual tumor. P values represent results from Wilcoxon rank-sum test; values < 0.05 were considered significant. (TIF) [file pone.0256139.s004.tif]
